# Material hardship, not household income, predicts impaired punishment learning: a computational reinforcement learning perspective

**DOI:** 10.3389/fpsyg.2025.1665380

**Published:** 2025-10-22

**Authors:** Zhen Wang, Xu He, Yunsheng Su, Laijun Bu, Yi Wang

**Affiliations:** ^1^Guangzhou Xinhua University, Dongguan, China; ^2^School of Public Health and Management, Guangzhou University of Chinese Medicine, Guangzhou, China; ^3^School of Psychology, South China Normal Univeristy, Guangzhou, China; ^4^School of Journalism and Communication, Jinan University, Guangzhou, China; ^5^School of Nursing, Guangdong Pharmaceutical University, Guangzhou, China; ^6^School of Journalism and Communication, Guangzhou University, Guangzhou, China

**Keywords:** material hardship, socioeconomic disadvantage, reinforcement learning, punishment learning, computational modeling

## Abstract

**Introduction:**

Socioeconomic disadvantage has been linked to neurocognitive alterations in reward and loss processing, which may contribute to adverse psychological outcomes. However, the mechanisms through which it influences reinforcement learning remain unclear.

**Methods:**

This study employed a Probabilistic Reversal Learning Task to examine how two distinct indicators of disadvantage—material hardship and low household income—affect reward and punishment-based learning in a sample of Chinese undergraduate students. Behavioral responses were analyzed through computational modeling within a reinforcement learning framework, estimating three key parameters: reward learning rate, punishment learning rate, and inverse temperature.

**Results:**

Results revealed that material hardship uniquely predicted individual differences in punishment learning rate, whereas household income showed no independent association with any of the model parameters.

**Discussion:**

The findings suggest that material hardship may specifically impair the ability to learn from negative outcomes. Furthermore, the study underscores the importance of distinguishing between material hardship and income-based adversity in research examining the cognitive impacts of socioeconomic disadvantage.

## 1 Introduction

In the field of cognitive science, reinforcement learning (RL) refers to a fundamental cognitive process by which individuals optimize their behavior based on environmental feedback (Shteingart and Loewenstein, 2014; Subramanian et al., 2022). This process operates via two dissociable pathways: (1) reward learning, where actions may be strengthened by positive outcomes (Daniel and Pollmann, 2014)—for example, an employee works harder after receiving a bonus—and (2) punishment learning, where behaviors may be modified to avoid adverse consequences, such as a driver slowing down after receiving a speeding ticket. Neuroscience research indicates that these pathways engage distinct neural substrates (Yacubian et al., 2006; Xue et al., 2013). Critically, extensive research has demonstrated that reward and punishment learning plays a crucial role in everyday decision-making (Lee et al., 2012), influencing behaviors across diverse contexts ranging from risk-taking (Marshall and Kirkpatrick, 2017) to social interactions (Heininga et al., 2017). This framework helps explain socioeconomic disparities in behavior; for instance, higher socioeconomic status has been linked to risky driving behaviors (Atombo et al., 2017), potentially because the punitive impact of fines is attenuated, disrupting the typical balance of punishment learning. While the behavioral and neural mechanisms of RL are well-documented, few studies investigate how individual differences, such as early-life experiences, influence these mechanisms. Investigating such factors may clarify the determinants of lifelong learning tendencies, thereby integrating cognitive models of decision-making with developmental psychology.

Given the established role of RL in daily life, a critical yet understudied question is how socioeconomic factors—particularly socioeconomic disadvantage—may shape these cognitive processes. Socioeconomic disadvantage exerts profound and far-reaching influences on human development, with measurable effects across multiple life domains including physical health (Torpy et al., 2007), mental well-being (Marbin et al., 2022), cognitive functioning (Mani et al., 2013), and economic decision-making (De Bruijn and Antonides, 2022). Notably, emerging neuroimaging evidence indicates that socioeconomic disadvantage may alter neurocognitive mechanisms relevant to RL, such as reward and loss processing. For example, White et al. (2022) found that a lower income-to-poverty ratio was associated with heightened neural responses to reward and loss cues during a passive avoidance task. Romens et al. (2015) demonstrated that increased neural activity during reward anticipation mediated the association between childhood poverty and depression symptoms, suggesting a potential neural pathway linking socioeconomic disadvantage to mental health outcomes. However, despite these advances, direct evidence on whether and how socioeconomic disadvantage modulates RL processes remains scarce. Addressing this gap could not only bridge cognitive science with developmental psychology but also inform interventions to mitigate the long-term behavioral impacts of socioeconomic disadvantage.

Over the past two decades, researchers have increasingly examined material hardship as a proximal measure of socioeconomic disadvantage (Gershoff et al., 2007; Thomas and Waldfogel, 2022). Unlike conventional income-based measures, material hardship reflects tangible deficits in meeting basic needs—such as food insecurity, unstable housing, and lack of medical care—providing a proximate framework to examine how acute scarcity shapes cognition and behavior (Beverly, 2001). Recent studies suggest that these experiences may influence economic decision-making, potentially altering how individuals evaluate risks and rewards. For example, He et al. (2024) reported that individuals with higher material hardship exhibited more loss-averse behavior in a mixed gambling task. Additionally, neuroimaging evidence demonstrates associations between material hardship and functional changes in frontal-limbic circuit (Chen et al., 2023), which is also a neural network critically involved in RL processes. These observations raise the possibility that material hardship, as a concrete manifestation of socioeconomic disadvantage, may directly modulate RL mechanisms, exacerbating maladaptive decision-making. By integrating material hardship into cognitive psychology, we can bridge the gap between macro-level socioeconomic factors and micro-level cognitive processes, ultimately clarifying how specific deprivation experiences shapes long-term behavior.

To empirically examine RL processes, researchers often employ probabilistic learning tasks (Koch et al., 2008; Daniel et al., 2020). In these paradigms, participants learn through trial and error to associate actions with probabilistically delivered rewards or punishments, thereby capturing adaptive learning under uncertainty (Soltani and Izquierdo, 2019). Computational RL models are then used to quantify the latent learning processes and individual differences (Schaaf et al., 2023). These models mathematically describe how individuals update their expectations based on feedback received, enabling the estimation of parameters reflecting distinct cognitive components. Key parameters include the learning rate, which determines how quickly expectations adjust to new feedback, and inverse temperature, which indicates the degree of randomness in decision-making (Katahira, 2015). Critically, while standard RL models apply a single learning rate to both reward and punishment outcomes, evidence from cognitive neuroscience research suggests dissociable neural substrates for these processes (Gueguen et al., 2021). This supports the use of a three-parameter model decoupling reward and punishment learning (den Ouden et al., 2013): the reward learning rate determines how rapidly expectations increase following gains, the punishment learning rate governs how rapidly expectations decrease following losses, and the inverse temperature parameter captures choice stochasticity.

Building upon this foundation and addressing the identified research gap, the current study employs a probabilistic reversal learning task coupled with the three-parameter computational RL model to empirically test whether socioeconomic disadvantage modulates core RL mechanisms. Specifically, we examine how two established indicators of disadvantage—material hardship and low household income—influence the efficiency of learning. These indicators are included as independent variables in regression analyses to assess their effects on two key computational parameters: the reward learning rate and the punishment learning rate. Based on emerging neurocognitive evidence linking socioeconomic adversity to heightened neural sensitivity to rewards and punishments (White et al., 2022), we hypothesized that greater socioeconomic disadvantage will be associated with elevated learning rates for both rewarding and punishing outcomes. This accelerated behavioral adaptation to feedback represents a potential cognitive mechanism through which socioeconomic disadvantage could shape long-term decision-making tendencies. By employing computational modeling within this well-established RL paradigm, our study moves beyond behavioral correlations to directly probe how disadvantage modulates these learning mechanisms, thereby illuminating cognitive pathways linking socioeconomic context to adaptive decision-making.

## 2 Materials and methods

### 2.1 Participants

The study protocol received ethical approval from the Research Ethics Committee of the author’s affiliated university. *A priori* power analysis using G*Power (Faul et al., 2007) indicated that a sample size of 84 provided 95% power to detect small effects (0.2) in multiple regression with up to 4 predictors at *α* = 0.05. A total of 100 first-year undergraduates were recruited from a public comprehensive university in China, where the average scores on the National College Entrance Examination (Gaokao) of admitted students fall within the mid-to-upper range nationally. Following exclusions for incomplete data or task accuracy below chance level, 95 participants (57 females, 38 males; aged 18–20 years, *M* ± *SD* = 18.44 ± 0.58) comprised the final sample. All participants reported normal or corrected-to-normal vision, and none reported a history of psychotropic medication use. Written informed consent was obtained prior to participation. Participants received ¥30–50 (approximately 5–6 USD) as compensation for their time.

### 2.2 Measures

*Material hardship*. Material hardship was assessed using the Chinese version of the Family Economic Hardship Questionnaire (Wang et al., 2010). The 4-item scale evaluates the frequency of material hardships across four domains: food insecurity, clothing affordability, access to entertainment, and housing stability. It has demonstrated strong psychometric properties in Chinese adolescent samples, with a Cronbach’s *α* of 0.84 in the original study and 0.83 in our sample. Participants rated each item on a 5-point Likert scale (1 = never, 5 = all the time). A composite score was calculated by averaging responses, with higher scores indicating greater material hardship.

*Household income*. Household income was self-reported using a 7-point ordinal scale: 1 (monthly income < ¥4,000 [≈5,060 USD]), 2 (¥4,000–7,999 [≈560–1,100 USD]), 3 (¥8,000–11,999 [≈1,100–1,680 USD]), 4 (¥12,000–15,999 [≈1,680–2,230 USD]), 5 (¥16,000–19,999 [≈2,230–2,800 USD]), 6 (¥20,000–39,999 [≈2,800–5,600 USD]), to 7 (≥¥40,000 [≈5,600 USD]), with lower scores indicating lower household income.

**Figure 1:**
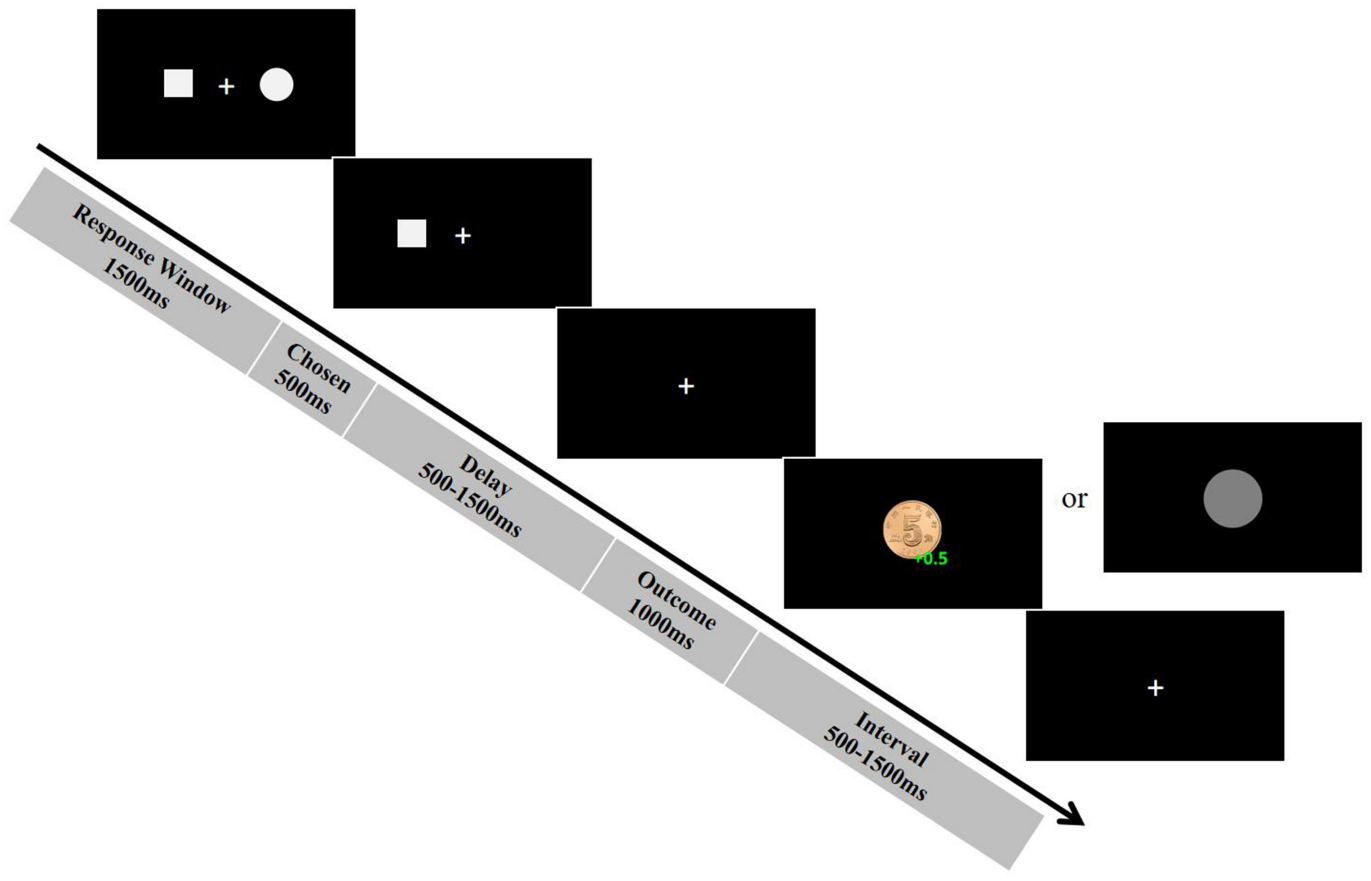
The procedure of a single trial in the probabilistic reversal learning task.

*Probabilistic reversal learning task*. Participants performed a computerized probabilistic reversal learning task (adapted from Gläscher et al., 2009) designed to measure reinforcement learning mechanisms under uncertainty. In this task, participants repeatedly selected between two visual stimuli—a square and a circle—presented simultaneously on each trial, with the goal of maximizing monetary rewards. They were explicitly informed that accumulated winnings would supplement their base compensation. Each trial followed a structured sequence: Following stimulus onset, participants had 1,500 ms to select one option; failure to respond within this window triggered an automatic random selection by the computer, with reaction time recorded as 1,500 ms. The chosen stimulus was then highlighted for 500 ms. After a variable delay (500–1,500 ms), the outcome (WIN ¥0.5 or LOSS ¥0) was displayed for 1,000 ms. Critically, stimulus-outcome contingencies were probabilistic: One stimulus was designated “correct” (75% probability of WIN; 25% probability of LOSS), while the other was “incorrect” (25% WIN; 75% LOSS). The “LOSS” outcome was coded as ¥0.00 (instead of a negative value) to avoid negative earnings throughout the task. This design was implemented to maintain participant motivation and engagement, and although the outcome is numerically zero, it is psychologically perceived as a loss relative to the winning outcome. A variable inter-trial interval (500–1,500 ms) followed, resulting in a mean trial duration of approximately 5,000 ms (see Figure 1 for schematic). To assess adaptive learning, the contingencies reversed randomly after 5 or 6 consecutive correct choices (this variability prevented anticipation of reversals). Participants needed to learn the new contingencies before another reversal could occur. Across the 60-trial task, up to 10 reversals were possible, with the total number of achieved reversals serving as a behavioral index of adaptive learning capacity. The computational model was fitted to each participant’s trial-by-trial choice data (i.e., which stimulus was selected on each trial), along with the corresponding outcomes (win or loss). Aggregate measures such as accuracy and reversal frequency were used solely as behavioral indices of task performance.

### 2.3 Computational modeling of reinforcement learning

While several computational frameworks exist for modeling reinforcement learning, we selected the three-parameter model (den Ouden et al., 2013) for its theoretical alignment with our research questions. This model distinguishes between reward and punishment learning rates, capturing dissociable mechanisms in belief updating. The model operates on a trial-by-trial basis. First, the prediction error ( ${\mathrm{PE}}_{t}$ ) is calculated as shown in Equation (1):


(1) $${\mathrm{PE}}_{t}=R_{t}-{\mathrm{EV}}_{t-1}$$

where $R_{t}$ is the outcome (scaled to 1 for win, −1 for loss) and ${\mathrm{EV}}_{t-1}$ is the expected value from the previous trial (initialized to 0 at *t* = 1). Then, the expected value for the chosen stimulus at trial *t* ( ${\mathrm{EV}}_{t}$ ) is updated using this prediction error according to Equation (2):


(2) $${\mathrm{EV}}_{t}={\mathrm{EV}}_{t-1}+\alpha \times {\mathrm{PE}}_{t}$$

This update is governed by separate learning rates ( α ) for positive and negative prediction errors; specifically, specifically, the reward learning rate ( $\alpha^{+}$ ) is applied when the ${\mathrm{PE}}_{t}>0$ , while the punishment learning rate ( $\alpha^{-}$ ) is applied when ${\mathrm{PE}}_{t}\leq 0$ . This follows the approach in the hBayesDM package (Ahn et al., 2017) for this class of models, where a prediction error ≤ 0 (outcome is worse than or equal to expectation) engages the punishment learning system for updating. Subsequently, the probability (P) of choosing options A and B is determined by a softmax function defined in Equation (3):


(3) $$P(A_{t})=\frac{1}{1+e^{-\beta \times ({\mathrm{EV}}_{A}-{\mathrm{EV}}_{B})}},P(B_{t})=1-P(A_{t})$$

where the inverse temperature parameter (β) governs the stochasticity of choices, with higher values indicating more deterministic, value-driven decision-making. Parameters ( $\alpha^{+}$ , $\alpha^{-}$ , β ) were estimated for each participant using a hierarchical Bayesian approach implemented in the hBayesDM package (Ahn et al., 2017) in R. This method was chosen because it provides more robust estimates by simultaneously modeling individual and group-level parameters, using the group distribution to constrain improbable individual estimates through partial pooling. Model parameters were estimated using Markov Chain Monte Carlo (MCMC) sampling, and convergence was successfully confirmed by R-hat values < 1.01.

### 2.4 Statistical analysis

Linear regression models examined how material hardship and household income independently predicted reward learning rate, punishment learning rate, and inverse temperature. Age and gender were included as covariates. Significance was evaluated at *p* < 0.05, with effect sizes reported as standardized coefficients (*b*). To ensure robustness, we also applied False Discovery Rate (FDR) correction for multiple comparisons across the three primary dependent variables (Benjamini and Hochberg, 1995). However, in interpreting the results, we focus on the pattern of effect sizes and their confidence intervals, as these provide more meaningful information than dichotomous significance testing alone.

We estimated three separate linear regression models. In each model, one of the computational parameters (reward learning rate, punishment learning rate, or inverse temperature) served as the dependent variable. The key independent variables of interest—material hardship and household income—were entered simultaneously into each model, along with the covariates of age and gender. This approach allowed us to test the unique association of each socioeconomic indicator with the learning parameters, while controlling for the other. In follow-up analyses, we examined the four subdomains of material hardship (food insecurity, clothing affordability, access to entertainment, and housing stability) in a separate regression model, with computational parameters as the dependent variable and household income, age, and gender included as covariates.

## 3 Results

**Figure 2:**
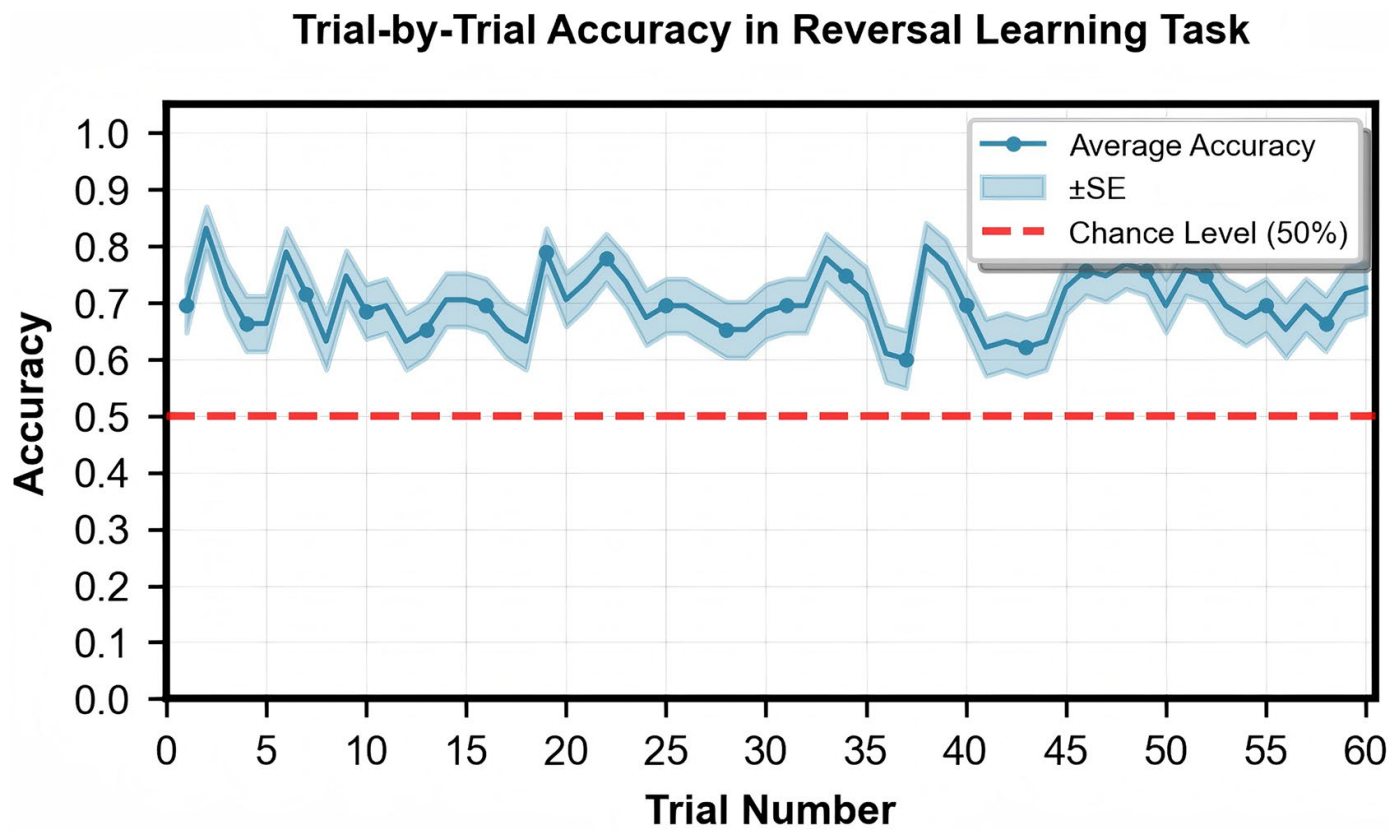
Trial-by-Trial accuracy in reversal learning task.

Participants completed 60 trials of the probabilistic reversal learning task, achieving a mean accuracy of 69.4% (*SD* = 6.7%, Table 1). A one-sample *t*-test confirmed that the overall accuracy (69.4%) was significantly above chance level (50%), *t*(94) = 28.22, *p* < 0.001, Cohen’s *d* = 2.895, indicating successful learning throughout the task (Figure 2 shows the trial-by-trial accuracy profile). The average number of successful reversals was 3.2 (*SD* = 1.5). Computational modeling using a hierarchical Bayesian approach estimated individual parameters for reward learning rate (*M* = 0.72, *SD* = 0.01), punishment learning rate (*M* = 0.54, *SD* = 0.09), and inverse temperature (*M* = 1.36, *SD* = 0.62). Model convergence was confirmed by R-hat values < 1.01.

**Table 1:** Descriptive statistics and bivariate correlations among study variables.

Variable	1	2	3	4	5	6	7	8
Mean	8.14	3.63	69.4%	603 ms	3.2	0.719	0.542	1.362
Standard deviation	3.65	1.62	6.7%	95 ms	1.5	0.008	0.089	0.619
1. Material hardship	—							
2. Household income	−0.417***	—						
3. Accuracy	−0.109	−0.041	—					
4. Reaction time	0.014	−0.004	−0.211*	—				
5. Reversal frequency	−0.209*	0.125	0.518***	−0.086	—			
6. Reward learning rate	−0.073	0.103	0.050	−0.048	0.283**	—		
7. Punishment learning rate	0.261*	−0.144	0.298**	−0.219*	−0.352***	−0.316**	—	
8. Inverse temperature	−0.077	0.072	0.579***	−0.189	0.513***	0.280**	0.085	—

*p < 0.05, **p < 0.01, ***p < 0.001.

Significant correlations emerged between task performance and model parameters: reward learning rate was positively associated with reversal frequency (*r* = 0.283, *p* = 0.006, 95% CI [0.089, 0.457]) but not significantly associated with accuracy (*r* = 0.050, *p* = 0.632, 95% CI [−0.124, 0.224]). While punishment learning rate showed positive correlation with accuracy (*r* = 0.298, *p* = 0.003, 95% CI [0.133, 0.438]), it was negatively associated with reversal frequency (*r* = −0.352, *p* < 0.001, 95% CI [−0.511, −0.180]). The inverse temperature parameter positively correlated with both accuracy (*r* = 0.579, *p* < 0.001, 95% CI [0.441, 0.699]) and reversal frequency (*r* = 0.513, *p* < 0.001, 95% CI [0.362, 0.649]).

**Figure 3:**
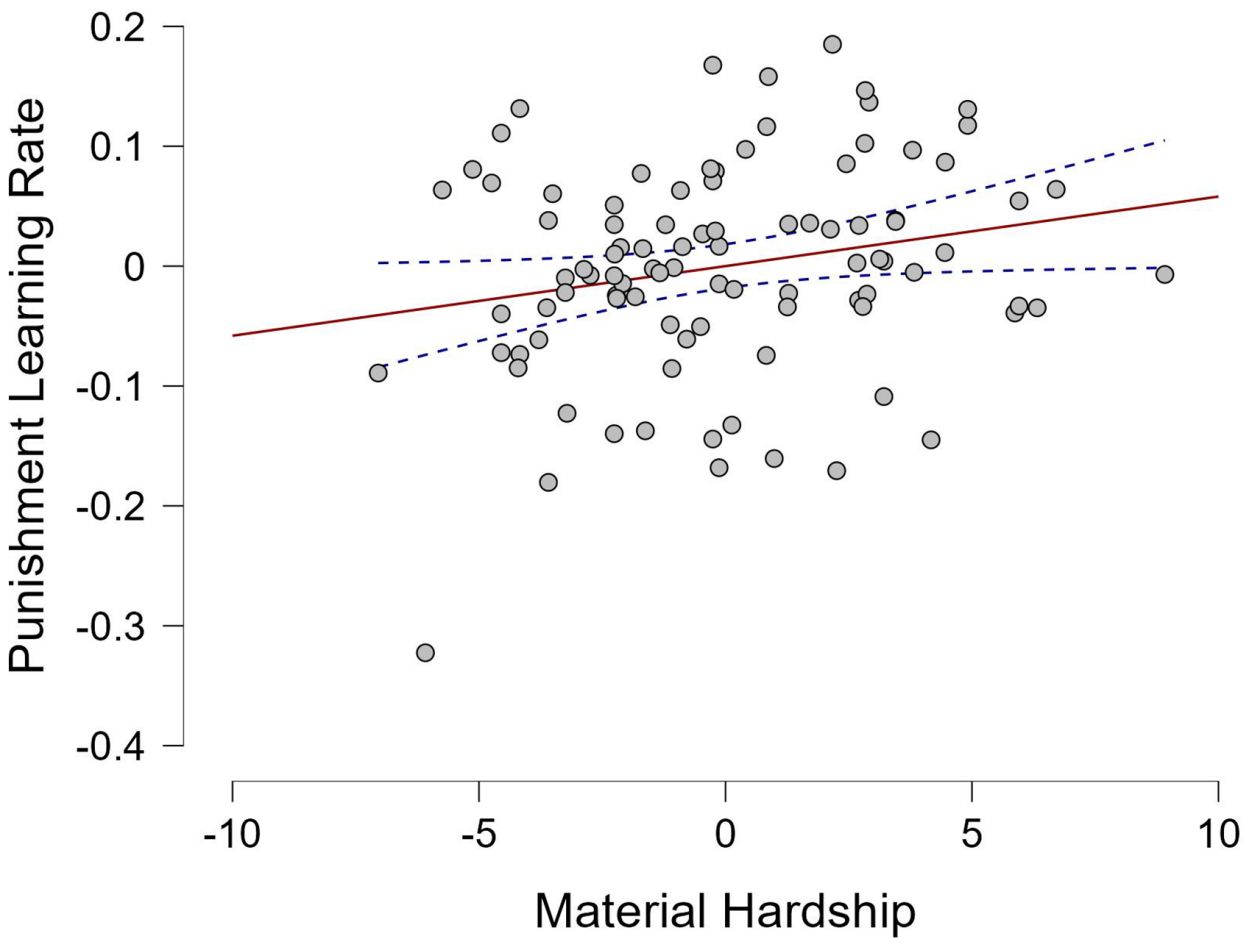
Partial association between material hardship and punishment learning rate after controlling for age, gender, and household income.

Bivariate analyses revealed that material hardship (*M* = 8.14, *SD* = 3.65) and household income (*M* = 3.63, *SD* = 1.62) were inversely correlated (*r* = −0.417, *p* < 0.001, 95% CI [−0.554, −0.251]). Material hardship correlated positively with punishment learning rate (*r* = 0.261, *p* = 0.011, 95% CI [0.080, 0.435], Figure 3) and negatively with reversal frequency (*r* = −0.209, *p* = 0.042, 95% CI [−0.382, −0.032]). Household income showed no significant correlations with reward learning rate (*r* = 0.103, *p* = 0.323, 95% CI [−0.068, 0.273]), punishment learning rate (*r* = −0.144, *p* = 0.164, 95% CI [−0.330, 0.042]), or inverse temperature (*r* = 0.072, *p* = 0.490, 95% CI [−0.125, 0.277]). Among hardship subdomains, housing instability (*M* = 2.38, *SD* = 1.40) showed the strongest correlation with reversal frequency (*r* = −0.218, *p* = 0.034, 95% CI [−0.408, −0.014]) and punishment learning rate (*r* = 0.274, *p* = 0.007, 95% CI [0.062, 0.460]).

Multiple regression analyses, which included both socioeconomic indicators while controlling for age and gender, revealed a distinct pattern of associations. Of primary theoretical interest, material hardship showed a positive association with punishment learning rate (*b* = 0.240, 95% CI [0.016, 0.464], uncorrected *p* = 0.036, FDR-corrected *p* = 0.108, Table 2). Although this association did not survive FDR correction, the medium effect size and the confidence interval excluding zero suggest a meaningful pattern consistent with the hypothesis that economic hardship sensitizes individuals to negative outcomes. In contrast, household income was not meaningfully associated with punishment learning rate (*b* = −0.046, *p* = 0.685) or any other model parameters (reward learning rate: *b* = 0.090, *p* = 0.439; inverse temperature: *b* = 0.038, *p* = 0.739). Material hardship itself demonstrated specificity, as it was not associated with reward learning rate (*b* = −0.034, *p* = 0.773) or inverse temperature (*b* = −0.067, *p* = 0.560). Neither age nor gender predicted any learning parameters (all *p* > 0.05). In exploratory follow-up regression models that examined hardship subdomains individually, only housing instability emerged as the unique predictor of punishment learning rate (*b* = 0.255, *p* = 0.022). Multicollinearity diagnostics indicated no concerns (all variance inflation factors < 1.3).

**Table 2:** Multiple regression analyses predicting reinforcement learning parameters.

Predictors	Reward learning rate			Punishment learning rate			Inverse temperature		
	*b*	95% CI	*p*	*b*	95% CI	*p*	*b*	95% CI	*p*
Material hardship	−0.034	[−0.263, 0.196]	0.773	0.240	[0.016, 0.464]	0.036	−0.067	[−0.296, 0.161]	0.560
Household income	0.090	[−0.140, 0.319]	0.439	−0.046	[−0.270, 0.178]	0.685	0.038	[−0.190, 0.267]	0.739
Age	−0.045	[−0.258, 0.167]	0.673	0.005	[−0.203, 0.212]	0.965	0.014	[−0.198, 0.226]	0.895
Gender	0.114	[−0.098, 0.325]	0.288	−0.051	[−0.257, 0.155]	0.624	−0.155	[−0.365, 0.056]	0.148

*b* = standardized beta coefficient. CI, confidence interval.

## 4 Discussion

This study directly addresses the critical gap concerning how socioeconomic disadvantage shapes RL mechanisms. By implementing a probabilistic reversal learning paradigm with a computational model that dissociates three core parameters—reward learning rate, punishment learning rate, and inverse temperature—we systematically evaluated the unique contributions of material hardship versus household income. Our findings reveal a targeted learning impairment: individuals experiencing material hardship, characterized by direct deprivation of basic needs, specifically exhibit heightened behavioral responsiveness to negative outcomes while maintaining intact reward processing. In contrast, household income demonstrated no significant relationship with any learning parameter. Among specific hardship subtypes, housing instability emerged as the strongest driver of this punishment sensitivity effect. Collectively, these results demonstrate how immediate deprivation experiences reconfigure fundamental learning mechanisms independently of financial constraints.

Our findings suggest a potential dissociation between socioeconomic indicators. Specifically, material hardship was associated with an elevated punishment learning rate, indicating heightened sensitivity to negative feedback, while reward learning remained unaffected. Although this association should be interpreted with caution as it did not survive strict correction for multiple comparisons, the observed effect size suggests a pattern worthy of further investigation. We propose that this hypersensitivity to punishment could become maladaptive in the current task by directly obstructing the acquisition of the latent task structure. Specifically, during learning phases which require ignoring occasional negative feedback to persist with the correct option, excessive reactivity to punishments causes premature abandonment of advantageous choices. Rather than tolerating probabilistic losses to maintain correct responding, they over-interpret negative outcomes as signals to switch strategies. This pattern reflects a failure to integrate feedback in a context-appropriate manner, ultimately obstructing the learning of latent task structure. The tendency to prioritize reactive switching over stable goal-directed behavior aligns with previous accounts of how adversity can bias decision-making under uncertainty (Lisi et al., 2025). Thus, socioeconomic disadvantage may recalibrate cognitive processes toward heightened reactivity to negative outcomes, perpetuating disadvantage cycles through maladaptive behavioral patterns.

Critically, our analyses demonstrate that material hardship, not household income, is the decisive socioeconomic factor driving alterations in punishment learning. While household income and material hardship are closely correlated, material hardship uniquely predicted both heightened punishment learning rates and poorer behavioral adaptation (i.e., reduced reversals). This dissociation aligns with longitudinal evidence showing material hardship independently predicts cognitive deficits beyond income effects (Daniel et al., 2024). We propose this occurs because immediate hardship generates perceived stress (Huang et al., 2021), which disproportionately overburdens neurocognitive systems governing threat response. Consequently, individuals become hyper-responsive to losses at the expense of adaptive flexibility. This pattern supports theoretical frameworks positing that distal variables shape the current life situation (Martin and Martin, 2002). Future research should prioritize measuring direct adversity experiences—such as unstable housing—as the critical pathways connecting socioeconomic disadvantage to cognitive changes.

Examining the subdomains of material hardship more closely, we found that housing instability emerged as the strongest predictor of impaired punishment learning. Longitudinal research showed that housing instability had a stronger effect on cognitive development than child maltreatment, poverty, and other risks (Fowler et al., 2015). A scoping review highlighted cognitive impairment as both a risk factor for and a consequence of homelessness (Stone et al., 2019). Unlike other financial pressures, housing insecurity uniquely compromises fundamental safety needs, keeping individuals in survival-mode cognition. Neuroimaging evidence confirms such adversity amplifies amygdala reactivity to stress (Tottenham, 2009), which partly explaining our findings. Critically, interventions that stabilizing housing—like housing vouchers—showed measurable psychological benefits (Finnie et al., 2022), making housing stability interventions a highly effective policy approach to reduce harmful cognitive effects linked to socioeconomic disadvantage.

Our identification of material hardship—particularly housing instability—as a primary mechanism driving maladaptive punishment learning necessitates structural policy interventions. Critically, approaches focused exclusively on income supplementation might be less effective in addressing the cognitive consequences of direct deprivation experiences. Effective solutions must instead target the tangible manifestations of material hardship through comprehensive social safety nets. These should include: (1) housing stabilization programs with eviction protection, (2) expanded food assistance, (3) universal healthcare access, and (4) guaranteed utility support. Such interventions directly reduce the chronic stress and perceived scarcity stemming from unmet basic needs—precisely the mechanism through which hardship amplifies neural sensitivity to negative outcomes in our study. By ensuring environmental stability, these policies create conditions conducive to neurocognitive recovery. As our findings demonstrate that secure housing specifically mitigates punishment hypersensitivity, prioritizing these multi-faceted supports will foster improved learning flexibility and adaptive decision-making in disadvantaged communities, ultimately disrupting cycles of socioeconomic disadvantage.

While this study advances our understanding of how socioeconomic disadvantage shapes learning, several limitations should be acknowledged. First, the cross-sectional design limits causal inference. Future longitudinal research should track how socioeconomic disadvantage influence learning mechanisms across development and examine whether interventions can modify these pathways. Second, our participant sample limits generalizability. Replication studies with more diverse populations and age groups are needed, particularly in understanding how socioeconomic disadvantage affects neurodevelopment in children and adolescents. Third, our computational modeling approach employed a parsimonious three-parameter model that dissociates reward and punishment learning rates. While this model choice was appropriate for our sample size, it does not capture all aspects of reinforcement learning, such as separate scaling parameters for reward and punishment sensitivity. Future studies with larger samples could employ more complex models to provide a more comprehensive account. Fourth, although computational modeling provides relatively precise parameter estimates, it cannot fully capture complex cognitive processes. Future work should therefore combine computational modeling with neuroimaging techniques, in order to map model-derived cognitive processes to their neural substrates and identify how hardship exposure affects neural systems underlying RL. Finally, future intervention research should empirically evaluate cognitive and behavioral strategies specifically designed to mitigate maladaptive patterns in punishment learning among disadvantaged populations—strategies that directly target the neurocognitive mechanisms identified here—with rigorous measurement of their efficacy in disrupting cycles of socioeconomic disadvantage.

## Data Availability

The datasets presented in this study can be found in online repositories. The names of the repository/repositories and accession number(s) can be found at: https://osf.io/zchxy/?view_only=88519feb52e244298b3c2dd0b225d8f0.

## References

[ref1] Ahn W.-Y. Haines N. Zhang L. (2017). Revealing neurocomputational mechanisms of reinforcement learning and decision-making with the hBayesDM package. Comput. Psychiatry 1:24. doi: 10.1162/CPSY_a_00002, PMID: 29601060 PMC5869013

[ref2] Atombo C. Wu C. Tettehfio E. O. Agbo A. A. (2017). Personality, socioeconomic status, attitude, intention and risky driving behavior. Cogent Psychol. 4:1376424. doi: 10.1080/23311908.2017.1376424

[ref3] Benjamini Y. Hochberg Y. (1995). Controlling the false discovery rate: a practical and powerful approach to multiple testing. J. R. Stat. Soc. Ser. B Stat Methodol. 57, 289–300. doi: 10.1111/j.2517-6161.1995.tb02031.x

[ref4] Beverly S. G. (2001). Measures of material hardship: rationale and recommendations. J. Poverty 5, 23–41. doi: 10.1300/J134v05n01_02

[ref5] Chen C. Wang Z. Cao X. Zhu J. (2023). Exploring the association between early exposure to material hardship and psychopathology through indirect effects of fronto-limbic functional connectivity during fear learning. Cereb. Cortex 33, 10702–10710. doi: 10.1093/cercor/bhad320, PMID: 37689831

[ref6] Daniel R. Pollmann S. (2014). A universal role of the ventral striatum in reward-based learning: evidence from human studies. Neurobiol. Learn. Mem. 114, 90–100. doi: 10.1016/j.nlm.2014.05.002, PMID: 24825620 PMC4143465

[ref7] Daniel R. Radulescu A. Niv Y. (2020). Intact reinforcement learning but impaired attentional control during multidimensional probabilistic learning in older adults. J. Neurosci. 40, 1084–1096. doi: 10.1523/JNEUROSCI.0254-19.2019, PMID: 31826943 PMC6989001

[ref8] Daniel G. Williams C. Lawrence A. Buckley K. Leonard D. Bernal D.. (2024). Income-based poverty and material hardship predict reduced cognitive performance in older American adults. Innov. Aging 8:1322. doi: 10.1093/geroni/igae098.4221

[ref9] De Bruijn E.-J. Antonides G. (2022). Poverty and economic decision making: a review of scarcity theory. Theor. Decis. 92, 5–37. doi: 10.1007/s11238-021-09802-7

[ref10] den Ouden H. E. M. Daw N. D. Fernandez G. Elshout J. A. Rijpkema M. Hoogman M.. (2013). Dissociable effects of dopamine and serotonin on reversal learning. Neuron 80, 1090–1100. doi: 10.1016/j.neuron.2013.08.030, PMID: 24267657

[ref11] Faul F. Erdfelder E. Lang A.-G. Buchner A. (2007). G*power 3: a flexible statistical power analysis program for the social, behavioral, and biomedical sciences. Behav. Res. Methods 39, 175–191. doi: 10.3758/BF03193146, PMID: 17695343

[ref12] Finnie R. K. C. Peng Y. Hahn R. A. Schwartz A. Emmons K. Montgomery A. E.. (2022). Tenant-based housing voucher programs: a community guide systematic review. J. Public Health Manag. Pract. 28, E795–E803. doi: 10.1097/phh.0000000000001588, PMID: 36194822 PMC9555591

[ref13] Fowler P. J. McGrath L. M. Henry D. B. Schoeny M. Chavira D. Taylor J. J.. (2015). Housing mobility and cognitive development: change in verbal and nonverbal abilities. Child Abuse Negl. 48, 104–118. doi: 10.1016/j.chiabu.2015.06.002, PMID: 26184055 PMC4593721

[ref14] Gershoff E. T. Aber J. L. Raver C. C. Lennon M. C. (2007). Income is not enough: incorporating material hardship into models of income associations with parenting and child development. Child Dev. 78, 70–95. doi: 10.1111/j.1467-8624.2007.00986.x, PMID: 17328694 PMC2835994

[ref15] Gläscher J. Hampton A. N. O’Doherty J. P. (2009). Determining a role for ventromedial prefrontal cortex in encoding action-based value signals during reward-related decision making. Cereb. Cortex 19, 483–495. doi: 10.1093/cercor/bhn098 18550593 PMC2626172

[ref16] Gueguen M. C. M. Lopez-Persem A. Billeke P. Lachaux J.-P. Rheims S. Kahane P.. (2021). Anatomical dissociation of intracerebral signals for reward and punishment prediction errors in humans. Nat. Commun. 12:3344. doi: 10.1038/s41467-021-23704-w, PMID: 34099678 PMC8184756

[ref17] He X. Qiu B. Deng Y. Wang Z. Cao X. Zheng X.. (2024). Material hardship predicts response bias in loss-averse decisions: the roles of anxiety and cognitive control. J. Psychol. 158, 309–324. doi: 10.1080/00223980.2023.2296946, PMID: 38227200

[ref18] Heininga V. E. Van Roekel E. Wichers M. Oldehinkel A. J. (2017). Reward and punishment learning in daily life: a replication study. PLoS One 12:e0180753. doi: 10.1371/journal.pone.0180753, PMID: 28976985 PMC5627895

[ref19] Huang Y. Heflin C. M. Validova A. (2021). Material hardship, perceived stress, and health in early adulthood. Ann. Epidemiol. 53, 69–75.e3. doi: 10.1016/j.annepidem.2020.08.017, PMID: 32949721 PMC7494502

[ref20] Katahira K. (2015). The relation between reinforcement learning parameters and the influence of reinforcement history on choice behavior. J. Math. Psychol. 66, 59–69. doi: 10.1016/j.jmp.2015.03.006

[ref21] Koch K. Schachtzabel C. Wagner G. Reichenbach J. R. Sauer H. Schlösser R. (2008). The neural correlates of reward-related trial-and-error learning: an fMRI study with a probabilistic learning task. Learn. Mem. 15, 728–732. doi: 10.1101/lm.1106408, PMID: 18832559

[ref22] Lee D. Seo H. Jung M. W. (2012). Neural basis of reinforcement learning and decision making. Annu. Rev. Neurosci. 35, 287–308. doi: 10.1146/annurev-neuro-062111-150512, PMID: 22462543 PMC3490621

[ref23] Lisi M. Michalek J. Hadfield K. Dajani R. Mareschal I. (2025). Effects of early adversity and war trauma on learning under uncertainty. Dev. Sci. 28:e70049. doi: 10.1111/desc.70049, PMID: 40778529 PMC12332969

[ref24] Mani A. Mullainathan S. Shafir E. Zhao J. (2013). Poverty impedes cognitive function. Science 341, 976–980. doi: 10.1126/science.1238041, PMID: 23990553

[ref25] Marbin D. Gutwinski S. Schreiter S. Heinz A. (2022). Perspectives in poverty and mental health. Front. Public Health 10:975482. doi: 10.3389/fpubh.2022.975482, PMID: 35991010 PMC9386343

[ref26] Marshall A. T. Kirkpatrick K. (2017). Reinforcement learning models of risky choice and the promotion of risk-taking by losses disguised as wins in rats. J. Exp. Psychol. Anim. Learn. Cogn. 43, 262–279. doi: 10.1037/xan0000141, PMID: 29120214 PMC5682951

[ref27] Martin P. Martin M. (2002). Proximal and distal influences on development: the model of developmental adaptation. Dev. Rev. 22, 78–96. doi: 10.1006/drev.2001.0538

[ref28] Romens S. E. Casement M. D. McAloon R. Keenan K. Hipwell A. E. Guyer A. E.. (2015). Adolescent girls’ neural response to reward mediates the relation between childhood financial disadvantage and depression. Child Psychol. Psychiatry 56, 1177–1184. doi: 10.1111/jcpp.12410, PMID: 25846746 PMC4593710

[ref29] Schaaf J. V. Weidinger L. Molleman L. Van Den Bos W. (2023). Test–retest reliability of reinforcement learning parameters. Behav. Res. 56, 4582–4599. doi: 10.3758/s13428-023-02203-4, PMID: 37684495 PMC11289054

[ref30] Shteingart H. Loewenstein Y. (2014). Reinforcement learning and human behavior. Curr. Opin. Neurobiol. 25, 93–98. doi: 10.1016/j.conb.2013.12.004, PMID: 24709606

[ref31] Soltani A. Izquierdo A. (2019). Adaptive learning under expected and unexpected uncertainty. Nat. Rev. Neurosci. 20, 635–644. doi: 10.1038/s41583-019-0180-y, PMID: 31147631 PMC6752962

[ref32] Stone B. Dowling S. Cameron A. (2019). Cognitive impairment and homelessness: a scoping review. Health Soc. Care Commun. 27, e125–e142. doi: 10.1111/hsc.12682, PMID: 30421478 PMC6849546

[ref33] Subramanian A. Chitlangia S. Baths V. (2022). Reinforcement learning and its connections with neuroscience and psychology. Neural Netw. 145, 271–287. doi: 10.1016/j.neunet.2021.10.003, PMID: 34781215

[ref34] Thomas M. M. C. Waldfogel J. (2022). What kind of “poverty” predicts CPS contact: income, material hardship, and differences among racialized groups. Child Youth Serv. Rev. 136:106400. doi: 10.1016/j.childyouth.2022.106400, PMID: 35462724 PMC8972944

[ref35] Torpy J. M. Lynm C. Glass R. M. (2007). Poverty and health. JAMA 298:1968. doi: 10.1001/jama.298.16.1968, PMID: 17954551

[ref36] Tottenham N. (2009). A review of adversity, the amygdala and the hippocampus: a consideration of developmental timing. Front. Hum. Neurosci. 3:68. doi: 10.3389/neuro.09.068.2009, PMID: 20161700 PMC2813726

[ref37] Wang J. Li D. Zhang W. (2010). Adolescents’ family financial difficulty and social adaptation: coping efficacy of compensatory, mediation, and moderation effects. J. Beijing Norm Univ. (Soc. Sci.) 4, 22–32.

[ref38] White S. F. Nusslock R. Miller G. E. (2022). Low socioeconomic status is associated with a greater neural response to both rewards and losses. J. Cogn. Neurosci. 34, 1939–1951. doi: 10.1162/jocn_a_01821, PMID: 35061015

[ref39] Xue G. Xue F. Droutman V. Lu Z.-L. Bechara A. Read S. (2013). Common neural mechanisms underlying reversal learning by reward and punishment. PLoS One 8:e82169. doi: 10.1371/journal.pone.0082169, PMID: 24349211 PMC3859585

[ref40] Yacubian J. Gläscher J. Schroeder K. Sommer T. Braus D. F. Büchel C. (2006). Dissociable systems for gain- and loss-related value predictions and errors of prediction in the human brain. J. Neurosci. 26, 9530–9537. doi: 10.1523/JNEUROSCI.2915-06.2006, PMID: 16971537 PMC6674602

